# From Struggle to Strength: A Multicentric Study on How Public Policies for Celiac Disease Transform Lives

**DOI:** 10.3390/nu16172855

**Published:** 2024-08-26

**Authors:** Ana Luísa Falcomer, Claudia B. Pratesi, Eduardo Yoshio Nakano, Cláudia Chaves, Mohammad Rostami-Nejad, Morad Guennouni, Ayşegül Aksan, Jacques Pouchot, Winfried Häuser, Renata Puppin Zandonadi

**Affiliations:** 1 Department of Nutrition, School of Health Sciences, University of Brasilia, Brasilia 70910-900, Brazil; 2 College of Population Health, University of New Mexico, Albuquerque, NM 87131, USA; 3 Department of Statistics, University of Brasilia, Brasilia 70910-900, Brazil; nakano@unb.br; 4 ESSV, Centre for Studies in Education and Innovation (CI&DEI), Polytechnic University of Viseu, 3504-510 Viseu, Portugal; cchaves@essv.ipv.pt; 5 Celiac Disease and Gluten Related Disorders Research Center, Research Institute for Gastroenterology and Liver Diseases, Shahid Beheshti University of Medical Sciences, Tehran 1416634793, Iran; m.rostamii@gmail.com; 6 Science and Technology Team, Higher School of Education and Training, Chouaib Doukkali University of El Jadida, El Jadida 24000, Morocco; morad.guennouni@gmail.com; 7 Laboratory of Health Sciences and Technologies, Higher Institute of Health Sciences of Settat, Hassan First University of Settat, Settat 26000, Morocco; 8 Institute of Nutritional Science, Justus-Liebig University, 35392 Giessen, Germany; ayseguel.aksan@ernaehrung.uni-giessen.de; 9 Service de Médecine Interne, Hôpital Européen Georges Pompidou, Assistance Publique–Hôpitaux de Paris, Université Paris Cité, 75015 Paris, France; jacques.pouchot@egp.aphp.fr; 10 Medizinisches Versorgungszentrum für Schmerzmedizin und Seelische Gesundheit Saarbrücken—St. Johann Health Care Center Pain Medicine and Mental Health Saarbrücken, St. Johann Großherzog-Friedrich-Straße 44, 66111 Saarbrücken, Germany; winfriedhaeuser@googlemail.com

**Keywords:** regulations, gluten-free, celiac disease public policies, celiac disease questionnaire

## Abstract

This multicenter study aims to assess the impact of public policies (PPs) on the health-related quality of life (HRQoL) of individuals with celiac disease (CD) using the Celiac Disease Questionnaire (CDQ) and PPs for Celiac Disease Score (PPCDS). This cross-sectional exploratory study was conducted in four stages: first, standardizing data from countries using the CDQ; second, analyzing PPs aimed at CD patients; third, statistically examining these data; and fourth, associating HRQoL indicators with corresponding PPs. This study analyzed 15 CDQ assessments from 12 countries from 2007 to 2023. It found that comprehensive PPs positively correlated with HRQoL outcomes (Spearman correlation of 0.358). However, policies specifically targeting gluten-free meals and certification did not significantly improve HRQoL individually, suggesting they may be more effective when implemented together. Additionally, specialized health services did not notably reduce gastrointestinal symptoms, underscoring the necessity for improved patient education to enhance the effectiveness of these services. This study concludes that implementing and rigorously monitoring regulations to support CD patients is crucial for enhancing their HRQoL.

## 1. Introduction

Celiac disease (CD) is a chronic autoimmune enteropathy caused by the consumption of gluten by individuals with a genetic predisposition. Its worldwide prevalence is between 1% and 2% [1,2,3]. Inflammation of the small intestine and villous atrophy happens to celiacs due to the ingestion of gluten; therefore, the disease treatment consists of a completely gluten-free diet (GFD) [1,2].

The clinical manifestations of CD are classified as classic and non-classic [3,4]. The classic signs and symptoms of the disease are gastrointestinal-related, the most frequent of which are diarrhea, constipation, pain and bloating, flatulence, and weight loss [4,5]. In addition, CD can also present extraintestinal manifestations such as anemia, osteoporosis, recurrent mouth ulcers, chronic fatigue, depression, and dermatitis herpetiform [4,6,7].

Patients often report experiencing symptoms, but there are cases wherein individuals with CD are asymptomatic even when presenting with intestinal mucosal damage [8,9]. The wide range of manifestations and clinical profiles, combined with the complexity of diagnosing this condition, has led to a global underestimation of its prevalence [9,10,11].

The impact of celiac disease on the well-being of individuals can be significant, affecting various aspects such as physical health, mental well-being, social interactions, and overall daily functioning [3,12]. Several studies used the Celiac Disease Questionnaire (CDQ) by Haüser et al. in 2007, designed to assess health-related quality of life (HRQoL) in patients with celiac disease [13]. Along with determining HRQoL, evaluating the effectiveness of public policies (PPs) on CD outcomes is important since legislation is crucial in providing support and improving the welfare of CD patients [14,15].

Public regulations should cater to individuals’ daily challenges with CD, including access to safe dining options and gluten-free products [14,16]. It is crucial to understand the socioeconomic impact of the disease when assessing policy efficacy, as it can lead to financial burdens [14,17]. Policies should aim to provide financial assistance, insurance coverage for gluten-free products, and other forms of support [18].

Different models of financial support for people with CD can be found worldwide. For instance, in Italy and Argentina, the government has established a system through which adults with CD are entitled to an allowance to offset the higher cost of gluten-free foods [16,19], whereas, in Portugal and Australia, a tax deduction is available for people with CD to claim expenses related to gluten-free products [16,20]. These approaches alleviate financial strain and promote the adherence to a strict gluten-free diet [16].

Overall assessment of PPs on CD outcomes requires a multidimensional approach that considers broader societal, economic, and psychological factors affecting affected individuals’ HRQoL [21,22,23]. Recognizing the significance of PPs for CD patients and their influence on quality of life emphasizes the need to evaluate how a country’s regulations for CD can directly impact the well-being of individuals with this condition [16,24].

This study aims to assess the correlation between the HRQoL of individuals with CD, as evaluated by the CDQ [13], and the Public Policies for Celiac Disease Score (PPCDS) [25]. By examining these two measures, we can establish the extent to which PPs are linked to enhanced HRQoL for this population.

## 2. Materials and Methods

### 2.1. Study Design

**Figure 1:**
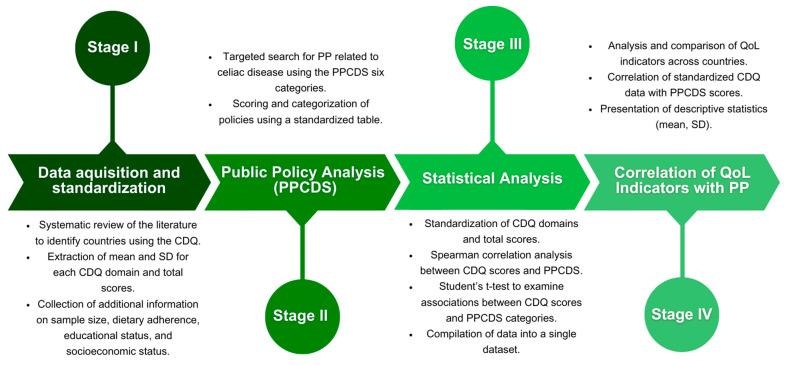
Comprehensive workflow of the multicentric quality of life and public policies for celiac disease research. CDQ: Celiac Disease Questionnaire; SD: standard deviation; PP: public policies; PPCDS: Public Policies for Celiac Disease Score.

This study is a multicenter, cross-sectional exploratory research conducted in four stages: (I) data acquisition and standardization from countries assessing the quality of life (QoL) of individuals with celiac disease (CD) using the CDQ; (II) analysis of public policies targeting people with CD in each participating country, spanning the years of CDQ application and the current study year, employing the PPCDS method; (III) statistical analysis of the gathered data; and (IV) correlation of QoL indicators across studied countries with their respective public policies aimed at the investigated population. A summary of the research process is presented in Figure 1.

To be included in this research, studies must have utilized the complete CDQ instrument for adult patients across various countries. No language or date restrictions were established. Studies that only evaluated children validated the instrument, or did not have openly published data were excluded from the study. Before excluding studies that did not have open data, we attempted contact with the corresponding author via email mentioned in the papers.

### 2.2. Data Collection and Screening Processes

The process of identifying countries that applied the CDQ instrument involved a systematic review of the published literature using the following terms, their mesh terms, and synonyms: “quality of life” AND (“celiac disease” OR “coeliac disease”) AND (“questionnaire” OR “instrument”) AND “adults” [26]. Our review was guided by the Preferred Reporting Items for Systematic Reviews and Meta-Analyses protocol, which enabled us to detect studies from different countries that could potentially meet the inclusion criteria [27].

After identifying eligible studies, the researchers extracted the mean and standard deviation of each CDQ domain and total scores for the celiac population in the respective country and year of data collection. Additionally, they gathered information on sample size, dietary adherence, educational status, and socioeconomic status from the CDQ assessment results.

The PPCDS evaluates the level of assistance provided by countries to their celiac populations through the assessment of six categories: the existence of regulations for GF industrial food and GF meals, specialized health service support, food allowance or financial incentives for individuals with celiac disease, gluten-free food certification, and support from celiac disease associations. Each category is scored on a scale from 0 to 1, where 1 represents the presence of relevant policies, and 0 indicates the absence of such policies. The total PPCDS score ranges from 0 to 6, with higher scores indicating more comprehensive and supportive public policies for individuals with celiac disease.

To assess PPs, two investigators conducted a targeted search for each country included in the QoL analysis, examining policy documents, government websites, and published literature on regulations, laws, and programs related to celiac disease from the same year as the instrument application and the current policies. The search strategy was performed using Google Search. It included combined keywords related to gluten-free products, meals, regulations, certifications, labeling, and terms associated with celiac disease, healthcare support, government assistance, and CD societies as proposed by the PPCDS original study [25].

The identified policies were categorized using a standardized table, with each policy marked as either ‘Yes’ or ‘No; for every policy marked as ‘Yes’’, 1 point was allocated. Whenever discrepancies in categorization arose between the two investigators, they discussed and reached a consensus.

For countries with non-English official languages, the searches were conducted in those languages with the assistance of the Google Translator tool. The CDQ scores and PPCDSs were then compiled into a single dataset, with each country represented as a data point.

### 2.3. Data Standardization and Quantitative Analysis

To allow for comparisons across countries, the CDQ domains and total scores, a 0–100 scale in two studies, were standardized to the instrument’s original 28–196 scale, where 196 represents the best possible quality of life [13]. The CDQ is a 28-item instrument that assesses four domains of quality of life: emotion, social, worries, and gastrointestinal. Each domain consists of 7 items scored on a 7-point Likert scale. As a result, the highest possible score for each domain is 49, and the total quality of life score is 196, which represents the sum of all the item scores.

To enable comparability between countries, we used the weighted mean and pooled standard deviation to present the CDQ findings consistently. This weighted comparison was necessary as data from two studies had been calculated for two groups and did not provide a final score [1,19].

After standardizing the CDQ data, we used the Spearman correlation to verify the relationship between the CDQ domains and total scores with the PPCDS. Additionally, a Student’s *t*-test was conducted to examine the strength and direction of the association between the CDQ domain and total scores and the six PPCDS categories. All analyses were performed using IBM SPSS Statistics for Windows [28]. Descriptive statistics, such as the mean and standard deviation, were presented to summarize the statistical analysis of the scores.

## 3. Results

Of the 21 studies identified through the systematic review, two were excluded for having validated the instrument but not applying it to the population [29,30]; two were excluded due to insufficient data [31,32]; and one was excluded for not thoroughly applying the CDQ instrument [33]. The remaining 15 studies, representing 12 different countries, were included in the final analysis, as they provided the necessary data for analyzing CDQ scores.

### 3.1. Studies Characteristics

The 15 studies included in the final analysis were conducted between 2007 and 2023 in 12 unique countries: Argentina (n = 1; 6.67%) [34], Australia (n = 1; 6.67%) [35], Brazil (n = 2; 13.33%) [36,37], France (n = 2; 13.33%) [21,38], Germany (n = 1; 6.67%) [13], Iran (n = 1; 6.67%) [39], Italy (n = 2; 13.33%) [3,19], Morocco (n = 1; 6.67%) [40], Portugal (n = 1; 6.67%) [41], Spain (n = 1; 6.67%) [42], Turkey (n = 1; 6.67%) [43] and the United Kingdom (n = 1; 6.67%) [1]. Notably, the dataset includes multiple studies from Brazil (2018 [36] and 2021 [37]), Italy (2011 [19] and 2013 [3]), and France (2014 [38] and 2022 [21]), allowing for an evaluation of temporal trends in these regions.

The geographical distribution of the studies spans five continents, with representation from South America (Argentina, Brazil), Oceania (Australia), Europe (France, Germany, Italy, Portugal, Spain, United Kingdom), Asia (Iran, Turkey), and Africa (Morocco). Most studies were conducted in European countries (n = 8; 53.33%).

In terms of sample characteristics, these studies involved 3982 celiac disease patients, with sample sizes ranging from 45 to 787 participants per study. The average age of the participants ranged from 29.83 to 49.0 years, with a higher proportion of females in all studies.

In some studies, the lack of demographic data on marital status, socioeconomic status, occupational status, education level, and dietary adherence to a gluten-free diet resulted in a subset of participant information being unavailable. This made it impossible to conduct further analysis across all the included studies.

Except for three studies that did not investigate dietary adherence [19,39,42], the celiac patients’ adherence to a gluten-free diet ranged from 50% to 100%. The studies used diverse methods to assess self-reported dietary adherence, such as a five-point Likert scale [3,34,36,37,41,43,44], a combination of the CDAT and GDF-S instruments [1], a 10-point visual analog scale [21], three-day food diary as well as self-report adherence [35], and a dichotomous inquiry [40].

Table 1 summarizes the countries’ CDQ domain scores and total scores, the PPCDSs, the studies’ sample sizes, and publication years. The table was ordered alphabetically and chronologically to optimize visualization in cases with multiple studies for a given country.

The total CDQ scores varied across countries, reflecting differences in the quality of life among individuals with celiac disease. The highest-scoring countries were Italy, the United Kingdom, and Germany. Italy ranks first and third with the best quality of life among celiac disease patients. The countries with the lowest scores are Iran, Morocco, and Portugal.

For countries that assessed the quality of life at two different time points, Italy and France showed an improvement, going from a score of 154.53 to 159.0 points and from 138.71 to 150.64, respectively. In contrast, a worsening in general QoL was observed in Brazil between 2018 and 2021.

Regarding public policies, Iran, Morocco, and Germany had the lowest scores on the PPCDS scale. Conversely, Italy in 2013, the United Kingdom, Australia, France, Spain, Argentina, and Portugal scored higher on the PPCDS. Details on the scores for each PPCDS category can be found in Table S1 in Supplementary Materials.

### 3.2. Public Policies for Celiac Disease Score (PPCDS)

**Figure 2:**
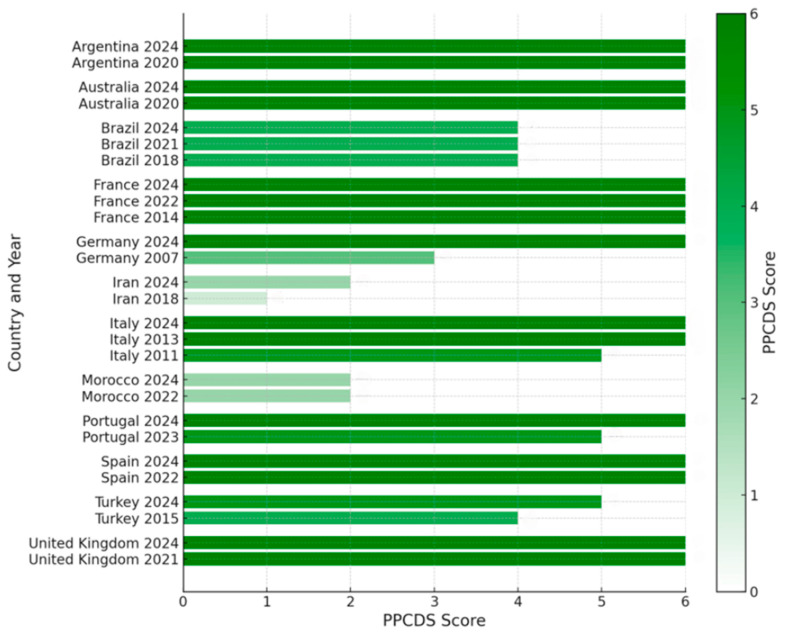
Comparative timeline of PPCDS across countries. PPCDS: Public Policies for Celiac Disease Score.

Figure 2 illustrates variations in the PPCDS across different countries and timelines. Argentina, Australia, France, Italy, and the United Kingdom consistently achieved the highest PPCDS of 6 over multiple years. In contrast, Brazil consistently scored 4 from 2018 to 2024. Germany improved from 3 in 2007 to 6 in 2024, and Turkey’s scores increased from 4 in 2015 to 5 in 2024. Although Iran’s PPCDS improved from 1 in 2018 to 2 in 2024, it remains categorized in the lower end, similar to Morocco, which maintained a score of 2 over the years. The data also highlight regional disparities, with European and Latin American countries achieving higher PPCDSs than Asian and African nations.

### 3.3. Association between HRQoL and PP

**Figure 3:**
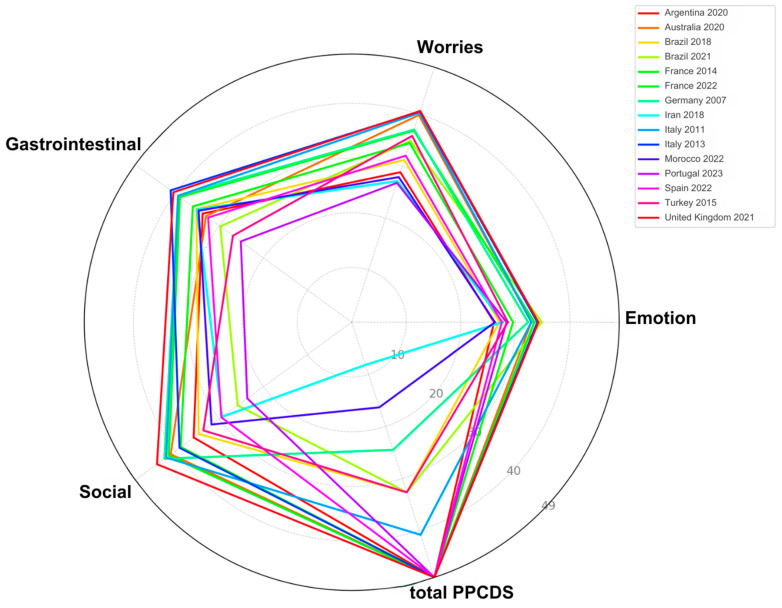
CDQ domain scores and total PPCDSs (normalized) by country and year. CDQ: Celiac Disease Questionnaire; PPCDS: Public Policies for Celiac Disease Score.

Figure 3 presents a spider chart that compares the CDQ domain scores—Emotion, Worries, Gastrointestinal, and Social—across 15 countries and years. Each axis represents one of the four domains, with scores normalized to a common scale from 0 to 49. The chart uses a diverse color palette based on the chromatic circle, ensuring clear differentiation between countries. This visualization allows for a comprehensive comparison of how different countries and years perform across these critical quality-of-life dimensions for individuals with celiac disease. The use of vibrant, non-repetitive colors enhances the chart’s readability, making it easier to identify patterns and contrasts in the domain scores across the analyzed countries.

The analysis revealed a positive association between PPCDS and CDQ, with a Spearman correlation score of 0.358. A positive association exists between most PPCDS items and CDQ domains (Table 2). However, the category of meal regulations was associated with reduced scores in the social and gastrointestinal CDQ domains and lower total CDQ scores (Table 2). Additionally, this PPCDS item was not associated with the worries CDQ domain. Furthermore, regulations concerning industrial food products and specialized health services did not show an association with the gastrointestinal CDQ domain. Lastly, gluten-free certification for manufactured meals was not associated with the emotion CDQ domain. Since all countries presented celiac disease associations, the correlation between this PP and CDQ cannot be evaluated.

## 4. Discussion

### 4.1. Insights on HRQoL Analysis

Both Brazilian studies observed that individuals who followed a strict GFD had higher overall QoL and by domains [36,37]. The same association was observed by the Argentinian [34], French [21,38], Portuguese [41], Turkish [43], British [1], and German [13] studies. Such observations also correlate with the low “worries” subscale scores since they are related to a GFD and, consequently, to regulations for GF products and their enforcement [16,45].

The substantial price gap between GF food and gluten-containing food likely contributes to the observed lower CDQ scores, as affordability concerns can negatively impact the mental well-being and social aspects of individuals with CD, which are directly reflected in the worries and social CDQ domains [46,47,48]. Studies have emphasized the need for governmental financial aid, as not having access to secure GF products and meals also burdens the health system due to disease advancement [24,49,50].

Strict adherence to the gluten-free diet can enhance physical and physiological well-being. Still, it may also put a strain on mental health and social aspects, as observed in the German celiac disease questionnaire [44]. Individuals may feel insecure about eating out, fear gluten cross-contamination, or be perceived as different for bringing home-cooked gluten-free meals when dining out [23,33]. Access to GF certification could reassure celiacs as it is a form of communication and transparency with consumers [51].

However, according to the latest French celiac disease questionnaire, an essential improvement in health-related quality of life was observed per additional year following the gluten-free diet [21]. The authors believe patients became habituated to managing the restrictive lifelong diet and found comfort in being part of celiac societies, resulting in less reported anxiety and fewer social difficulties the longer they had adhered to the diet [21,33].

Most European countries, with the exception of Portugal, scored higher in the CDQ (Table 1). However, it is essential to note that the assessment of HRQoL of Portuguese celiacs occurred during the COVID-19 pandemic [41]. The two studies that also collected data during the pandemic period were the Spanish and the Brazilian second study [37,42].

Portugal’s research obtained general sub-optimal scores, but it should be considered that 44.9% (n = 129) of the study’s participants did not follow a strict GFD [41]. Therefore, even though being at home could offer more trust in the secureness of the meal prepared, participants could have fallen into the temptation of eating gluten-containing food with family members, for instance [12,23,41].

According to the study performed in Brazil during the COVID-19 pandemic, Brazilians obtained higher scores in the social and worries domains, which could be related to the fact that 88.57% (n = 597) of the participants adhered to the dietary treatment [37]. The Spanish research also observed a higher score in the social item [42]. As a positive outcome, the pandemic period provided a sense of safety as meals were mainly prepared at home, and celiacs could avoid social events in places with gluten-containing food [37,52].

When analyzing the countries with HRQoL accessed more than once, France and Italy have both bettered their overall scores and subcategories [3,19,21,38]. Brazil obtained an overall score of 1.8 points lower and better in the emotion and worries categories than in the first assessment [36,37]. However, as mentioned, this second CDQ application occurred during the pandemic [37]. Following up on countries’ CD HRQoL periodically would be interesting so governments and health professionals can identify opportunities to improve celiac patient support [15,25].

### 4.2. Public Policies for Celiac Disease Score (PPCDS)

As evidenced in Figure 2, the European and Oceanian countries tend to have higher PPCDSs. These continents, alongside North America, have presented an increase in CD diagnoses in recent decades [25,53]. Conversely, the scores indicate that the African and Asian countries maintain relatively lower PPCDSs as in the 2019 assessment [25]. This is likely due to the lack of comprehensive population-based studies in regions such as Africa and Asia, which could explain the lower investment in patient support in those areas compared to other continents [53].

The exception is Turkey, an intercontinental country categorized as an Asian country for analytical purposes by the World Health Organization [54]. Turkey´s one-point improvement in the PPCDS between 2015 and 2024 is due to implementing a financial incentive policy for patients with CD, who are categorized today with a high score [25]. That indicates a national effort to support this population.

Additionally to Turkey, other countries have bettered their PPCDS since the period of the CDQ assessment. In 2007, Germany had a moderate PPCDS of 3 points, which was still higher than Iran and Morocco´s present scores. At the time, Germany presented regulations for gluten-free food products, specialized health services, and celiac society.

Since then, Germany has implemented policies related to partial financial aid through tax deductions for the extra costs associated with gluten-free foods if they exceed a certain percentage of the individual’s income. The UK, Spain, Italy, France, Australia, and Portugal already had PPs regarding food allowances or financial support.

The UK expanded the list of items in the GFD prescription provided through the National Health System. Argentina, Italy, and Spain augmented the allowances according to inflation and the cost of GF food and expanded the eligibility criteria. Portugal provided partial subsidies for GF food in 2023 and implemented a new policy giving tax deductions for the additional costs of medically prescribed GF food. Australia adjusted the process of claiming tax deductions for the additional costs of the GFD [20].

It is imperative to highlight that the financial assistance PP is, in all countries included in this study, not available in the whole nation´s territory [48,55]. As celiac disease cases continue to spike, there is an impending need for reevaluating patient care and expanding regulations to ensure patient security [15,56].

Although access to CD diagnostic tools may have increased, healthcare professionals’ need for knowledge and the diverse symptoms of this chronic condition still present challenges to suspecting and confirming the diagnosis [57,58]. Therefore, having CD-specialized healthcare is important to minimize delay in diagnosis and reduce exposure to gluten, which impacts QoL and life expectancy [59,60].

Nowadays, all countries included in this research have health centers with pathology-trained professionals. In addition, the records found of these centers were linked to universities, schools, and hospitals in all nations. That suggests that CD knowledge needs to be publicized to already-trained health professionals and beyond the academic sphere [57,58].

Regarding the lowest-scoring countries, as shown in Figure 2, Iran and Morocco´s scores suggest poorer support for individuals with CD in these countries than others. These findings align with [40].

According to the PPCDS data (Figure 2), despite the improvement in Iran´s score, it is still alongside Morocco, the only two countries in this study that lack formal national regulations for gluten-free food labeling. In these countries, industries and restaurants follow general food safety standards focused on quality and safety, but there are no specific gluten-free labeling requirements. This represents food insecurity and elevates worries for individuals with celiac disease [15,56].

Additionally, manufacturers who voluntarily include gluten information to appeal to health-conscious consumers and those with dietary restrictions frequently do not have exclusively gluten-free production [61]. As a result, the imported products certified as gluten-free are the most trustworthy options. Still, they are seen as a luxury since the products are hard to find and have elevated prices, making access to them difficult in Morocco and Iran [18,40].

Since gluten-free products cost more than traditional food items, maintaining a lifelong GFD can be onerous and a significant barrier to treatment adherence [49,62]. As previously documented, the cost disparity between gluten-containing products and gluten-free counterparts varies across countries [14,17]. For example, in Greece, gluten-free products can be 22–334% more expensive in supermarkets and 88–476% in pharmacies [63]. Meanwhile, in Morocco, gluten-free prices can be 115–1309% higher than regular versions [61].

Furthermore, the elevated cost of gluten-free products represents a significant barrier to adhering to the necessary treatment, and the GFD directly impacts the gastrointestinal domains and indirectly affects the emotional and social aspects of health-related quality of life [18,56,62].

### 4.3. Association between HRQoL and PPs

This study is the first to investigate the relationship between the HRQoL of individuals with CD and the PPs in place for this patient population. The findings reveal a positive correlation between the PPCDSs and the CDQ scores, suggesting that countries with more comprehensive public policies addressing celiac disease tend to have better overall quality of life outcomes for those with this condition.

While most PPCDS components positively correlated with various CDQ domains (Figure 3 and Table 2), indicating broad benefits for celiac patients across different aspects of life, an exception was observed in meal regulations. This specific policy category was associated with lower scores in the social and gastrointestinal domains of the CDQ and the overall CDQ scores.

These meal regulations may not be fully enforced, or their implementation may not effectively address the social and gastrointestinal concerns of individuals with celiac disease. Producing gluten-free meals in professional kitchens that also handle gluten-containing foods can pose significant challenges [64,65,66]. As a result, individuals with celiac disease often opt to dine in exclusively gluten-free restaurants [62,67,68].

While the meals may be safe, celiacs have reported feeling socially isolated and judged when inviting family members and friends to eat in a 100% gluten-free establishment, which can negatively impact their social experiences [12,23,33]. Hence, having laws to guide GF meal production does not mean that the policies are being followed or that celiac consumers will trust the restaurants; this result suggests that this PP alone will not make people with CD more confident about dining out [23,33,51].

Similarly, the presence of GF certification for manufactured meals did not show a significant positive association in the worries domain of the CDQ, indicating that, while this certification is essential and could be effective, it alone may not be sufficient to alleviate the concerns and fears experienced by individuals with CD [23,33]. Considering that consumers’ trust in certifications is related to the perception of credibility in the food production process, the simultaneous implementation of meal regulations and GF certification could be more effective in attenuating concerns about celiacs [51].

Moreover, the availability of specialized health services did not correlate with improvements in the gastrointestinal domain of the CDQ. This absence of association could have been due to the reduced number of specialized health centers in each country. Therefore, celiacs are still attended to, and cared for by health professionals who are not trained in CD [57,58].

### 4.4. Study Limitations

This study’s cross-country comparisons between developed and developing economies with varying public policies may limit the generalizability of its findings to all countries globally. They may not represent continental-level assistance for celiac patients. Additionally, the HRQoL data included only some CDQ assessments made to date, as the researchers could not assess data from three articles that may have met the inclusion criteria despite attempts to contact the authors via email and social media.

Furthermore, the PPCDSs may not fully capture the nuances of how PPs are implemented and enforced across diverse cultural and socioeconomic contexts. Additionally, since some of the policies included in the PPCDS are only regional rather than national, some participants in the CDQ may have access to these policies, which could have diminished the observed association between public policies and quality of life.

## 5. Conclusions

The findings of this study suggest that PPs designed to address the needs of individuals with CD are associated with better HRQoL outcomes. By highlighting the complex relationship between specific policy domains and various aspects of well-being, this research underscores the importance of a multidimensional approach to policymaking and implementation to support the CD community effectively. Monitoring the enforcement of these regulations, expanding them to all national territories, and educating health professionals to assist celiac patients are necessary steps. Future research should further explore the nuances of policy implementation and additional factors that may influence the QoL for those living with CD. In summary, monitoring the QoL of these individuals over time would provide valuable insights to governments and policymakers.

## Figures and Tables

**Table 1:** CDQ scores and PPCDSs by country and year.

	Year	n	PPCDS			CDQ		
				Emotion	Social	Worries	Gastrointestinal	Total
				Mean (SD)	Mean (SD)	Mean (SD)	Mean (SD)	Mean (SD)
Argentina [34]	2020	171	6	26.07 (10.38)	35.80 (9.25)	28.82 (10.11)	33.77 (9.24)	124.14 (32.44)
Australia [35]	2020	45	6	32.90 (0.99)	41.00 (6.12)	39.80 (0.79)	33.00 (0.88)	147.00 (3.31)
Brazil [36]	2018	450	4	27.06 (10.08)	34.67 (7.08)	31.17 (8.54)	35.17 (6.23)	128.06 (27.08)
Brazil [37]	2021	674	4	34.81 (8.42)	25.82 (8.87)	34.86 (10.25)	29.77 (10.75)	125.26 (32.02)
France [38]	2014	211	6	29.55 (9.20)	38.75 (9.91)	34.43 (9.62)	35.98 (8.07)	138.71 (30.91)
France [21]	2022	787	6	33.46 (8.82)	41.44 (8.40)	36.82 (8.82)	38.92 (7.98)	150.64 (20.16)
Germany [44]	2007	446	3	32.30 (8.50)	42.40 (7.10)	37.00 (8.80)	39.30 (7.10)	151.10 (25.20)
Iran [39]	2018	81	1	27.64 (10.81)	29.37 (10.72)	27.11 (10.38)	35.06 (9.76)	119.18 (34.00)
Italy [19]	2011	187	5	32.92 (7.62)	42.09 (6.75)	40.24 (51.34)	39.31 (5.82)	154.53 (20.86)
Italy [3]	2013	171	6	34.00 (8.00)	43.00 (7.00)	40.00 (8.00)	41.00 (7.00)	159.00 (24.00)
Morocco [40]	2022	112	2	26.23 (4.68)	31.76 (9.69)	25.03 (8.36)	34.67 (6.72)	117.73 (24.61)
Portugal [41]	2023	234	6	28.35 (7.60)	23.03 (9.53)	26.77 (8.78)	25.12 (8.81)	103.28 (31.15)
Spain [42]	2022	92	6	27.48 (4.78)	40.23 (5.84)	30.79 (5.72)	32.53 (7.76)	131.03 (24.10)
Turkey [43]	2015	205	4	28.60 (9.00)	34.00 (8.10)	28.00 (8.50)	34.20 (8.30)	124.80 (28.10)
United Kingdom [1]	2021	116	6	34.20 (5.57)	44.10 (6.32)	40.40 (6.78)	37.85 (7.63)	156.55 (21.77)

SD: Standard Deviation; PPCDS: Public Policies for Celiac Disease Score; CDQ: Celiac Disease Questionnaire.

**Table 2:** Relationship between CDQ scores and PPCDS categories.

		Yes	No	*p* ^2^
		Mean (SD) ^1^	Mean (SD) ^1^	
Regulations concerning industrial food products	n	3789	193	
	Emotion	31.54 (8.60)	26.82 (7.85)	0.000
	Social	36.18 (8.11)	30.76 (10.13)	0.000
	Worries	34.54 (14.33)	25.90 (9.26)	0.000
	Gastrointestinal	35.24 (8.20)	34.83 (8.13)	0.496
	Total	137.55 (26.58)	118.34 (28.92)	0.000
Regulations relating to meals	n	2951	1031	
	Emotion	31.54 (8.65)	30.65 (8.32)	0.003
	Social	35.01 (8.34)	38.49 (7.88)	0.000
	Worries	34.26 (8.99)	33.72 (23.24)	0.466
	Gastrointestinal	34.44 (8.48)	37.45 (7.34)	0.000
	Total	135.31 (27.00)	140.36 (25.79)	0.000
Specialized health	n	3901	81	
service support	Emotion	31.39 (8.51)	27.64 (10.88)	0.003
	Social	36.05 (8.16)	29.37 (10.79)	0.000
	Worries	34.27 (14.20)	27.11 (10.45)	0.000
	Gastrointestinal	35.23 (8.16)	35.06 (9.82)	0.880
	Total	136.98 (26.52)	119.18 (34.21)	0.000
Food allowance and/or	n	2014	1968	
financial incentive	Emotion	31.58 (8.28)	31.04 (8.84)	0.046
	Social	38.82 (8.28)	32.94 (8.16)	0.000
	Worries	35.31 (17.63)	32.91 (9.27)	0.000
	Gastrointestinal	36.30 (7.82)	34.12 (8.57)	0.000
	Total	142.07 (24.54)	131.03 (28.73)	0.000
Gluten-free certification for manufactured meals	n	2219	1763	
	Emotion	31.31 (8.35)	31.32 (8.86)	0.954
	Social	38.38 (8.26)	32.81 (8.30)	0.000
	Worries	34.63 (16.99)	33.48 (9.44)	0.007
	Gastrointestinal	36.10 (7.87)	34.11 (8.66)	0.000
	Total	140.48 (24.88)	131.75 (29.05)	0.000
Celiac disease	n	3982	0	
Associations ^3^	Emotion	31.31 (8.56)	-	-
	Social	35.91 (8.22)	-	-
	Worries	34.12 (14.13)	-	-
	Gastrointestinal	35.22 (8.20)	-	-
	Total	136.61 (26.69)	-	-

^1^ Weighted mean and Pooled Standard Deviation; ^2^ Independent Student *t* test; ^3^ All countries presented celiac disease associations.

## Data Availability

Data are contained within the article and Supplementary Materials.

## Supplementary Materials

The following supporting information can be downloaded at https://www.mdpi.com/article/10.3390/nu16172855/s1, Table S1: Public Policies for Celiac Disease Score distribution per country.

## References

[1] Dimidi E., Kabir B., Singh J., Ageridou A., Foster C., Ciclitira P., Dubois P., Whelan K. (2021). Predictors of adherence to a gluten-free diet in celiac disease: Do knowledge, attitudes, experiences, symptoms, and quality of life play a role?. Nutrition.

[2] Mulder C.J.J., Van Wanrooij R.L.J., Bakker S.F., Wierdsma N., Bouma G. (2013). Gluten-free diet in gluten-related disorders. Dig. Dis..

[3] Marchese A., Klersy C., Biagi F., Balduzzi D., Bianchi P.I., Trotta L., Vattiato C., Zilli A., Rademacher J., Andrealli A. (2013). Quality of life in coeliac patients: Italian validation of a coeliac questionnaire. Eur. J. Intern. Med..

[4] Caio G., Volta U., Sapone A., Leffler D.A., De Giorgio R., Catassi C., Fasano A. (2019). Celiac disease: A comprehensive current review. BMC Med..

[5] Paavola A., Kurppa K., Ukkola A., Collin P., Lähdeaho M.L., Huhtala H., Mäki M., Kaukinen K. (2012). Gastrointestinal symptoms and quality of life in screen-detected celiac disease. Dig. Liver Dis..

[6] Tovoli F. (2015). Clinical and diagnostic aspects of gluten related disorders. World J. Clin. Cases.

[7] Elli L., Branchi F., Tomba C., Villalta D., Norsa L., Ferretti F., Roncoroni L., Bardella M.T. (2015). Diagnosis of gluten related disorders: Celiac disease, wheat allergy and non-celiac gluten sensitivity. World J. Gastroenterol..

[8] Itzlinger A., Branchi F., Elli L., Schumann M. (2018). Gluten-Free Diet in Celiac Disease—Forever and for All?. Nutrients.

[9] Husby S., Koletzko S., Korponay-Szabó I., Kurppa K., Mearin M.L., Ribes-Koninckx C., Shamir R., Troncone R., Auricchio R., Castillejo G. (2020). European Society Paediatric Gastroenterology, Hepatology and Nutrition guidelines for diagnosing coeliac disease 2020. J. Pediatr. Gastroenterol. Nutr..

[10] Singh P., Arora A., Strand T.A., Leffler D.A., Catassi C., Green P.H., Kelly C.P., Makharia G.K. (2018). Global Prevalence of Celiac Disease: Systematic Review and Meta-analysis. Clin. Gastroenterol. Hepatol..

[11] Mustalahti K., Catassi C., Reunanen A., Fabiani E., Heier M., McMillan S., McMillan S., Murray L., Metzger M.-H., Gasparin M. (2010). The prevalence of celiac disease in Europe: Results of a centralized, international mass screening project. Ann. Med..

[12] Lee A.R., Wolf R., Contento I., Verdeli H., Green P.H.R.R. (2015). Coeliac disease: The association between quality of life and social support network participation. J. Hum. Nutr. Diet..

[13] Haüser W., Gold J., Stallmach A., Caspary W.F., Stein J. (2007). Development and Validation of the Celiac Disease Quality of Life Measure for Adult Patients with Celiac Disease. J. Clin. Gastroenterol..

[14] Linton M.J., Jones T., Owen-Smith A., Payne R.A., Coast J., Glynn J., Hollingworth W. (2018). Breaking bread: Examining the impact of policy changes in access to state-funded provisions of gluten-free foods in England. BMC Med..

[15] Siminiuc R., Turcanu D. (2022). Food security of people with celiac disease in the Republic of Moldova through prism of public policies. Front. Publich Health..

[16] Pinto-Sanchez M.I., Verdu E.F., Gordillo M.C., Bai J.C., Birch S., Moayyedi P., Bercik P. (2015). Tax-deductible provisions for gluten-free diet in Canada compared with systems for gluten-free diet coverage available in various countries. Can. J. Gastroenterol. Hepatol..

[17] Long K.H., Wagie A.E., Iii L.J.M., Lahr B.D., Van Dyke C.T., Murray J.A. (2010). The economics of coeliac disease: A population-based study. Aliment. Pharmacol. Ther..

[18] Nadal J., Ferreira S.M.R., da Costa I.B., Schmidt S.T. (2013). The principle of human right to adequate food and celiac disease: Advancements and challenges. Demetra: Food Nutr. Health.

[19] Zampieron A., Daicampi C., Martin A., Buja A. (2011). Quality of life in adult celiac disease in a mountain area of Northeast Italy. Gastroenterol. Nurs..

[20] National Celiac Association (2024). Tax Deductions for Gluten-Free Food. https://nationalceliac.org/tax-deductions-for-gluten-free-food/.

[21] Enaud R., Tetard C., Dupuis R., Laharie D., Lamireau T., Zerbib F., Rivière P., Shili-Mismoud S., Poullenot F. (2022). Compliance with Gluten Free Diet Is Associated with Better Quality of Life in Celiac Disease. Nutrients.

[22] Shamir R., Heyman M.B., Koning F., Wijimenga C., Gutierrez-Achury J., Catassi C., Gatti S., Fasano A., Discepolo V., Korponay-Szabó I.R. (2014). Celiac Disease: Past, Present, and Future Challenges. J. Pediatr. Gastroenterol. Nutr..

[23] Bacigalupe G., Plocha A. (2015). Celiac is a social disease: Family challenges and strategies. Fam. Syst. Health.

[24] Gatti S., Rubio-Tapia A., Makharia G., Catassi C. (2024). Patient and Community Health Global Burden in a World With More Celiac Disease. Gastroenterology.

[25] Falcomer A.L., Luchine B.A., Gadelha H.R., Szelmenczi J.R., Nakano E.Y., Farage P., Zandonadi R.P. (2020). Worldwide public policies for celiac disease: Are patients well assisted?. Int. J. Public Health.

[26] Falcomer A.L., de Lima B.R., Farage P., Fabris S., Ritter R., Raposo A., Teixeira-Lemos E., Chaves C.B., Zandonadi R.P. (2024). Enhancing life with celiac disease: Unveiling effective tools for assessing health-related quality of life. Front. Immunol..

[27] Moher D., Shamseer L., Clarke M., Ghersi D., Liberati A., Petticrew M., Shekelle P., Stewart L.A., PRISMA-P Group (2015). Preferred reporting items for systematic review and meta-analysis protocols (PRISMA-P) 2015 statement. Syst. Rev..

[28] IBM Corp (2016). SPSS: Statistical Package for the Social Sciences.

[29] Lobão C., Gonçalves R., Baltazar R.M. (2013). Desenvolvimento da versão portuguesa do celiac disease questionnaire. Rev. Int. Cienc. Sociales..

[30] Häuser W., Gold J., Stein J., Caspary W.F., Stallmach A. (2006). Health-related quality of life in adult coeliac disease in Germany: Results of a national survey. Eur. J. Gastroenterol. Hepatol..

[31] Szőcs H., Horváth Z., Vizin G. (2021). The mediating role of shame in the relationship between stigma and quality of life in patients with celiac disease. Orv. Hetil..

[32] Schiepatti A., Maimaris S., De Queiros C., Archela M., Rusca G., Costa S., Biagi F. (2021). Long-Term Adherence to a Gluten-Free Diet and Quality of Life of Celiac Patients After Transition to an Adult Referral Center. Dig. Dis. Sci..

[33] Zysk W., Glabska D., Guzek D., Głąbska D., Guzek D. (2018). Social and Emotional Fears and Worries Influencing the Quality of Life of Female Celiac Disease Patients Following a Gluten-Free Diet. Nutrients.

[34] Selleski N., Zandonadi R.P., Milde L.B., Gandolfi L., Pratesi R., Häuser W., Uenishi R.H., Nakano E.Y., Pratesi C.B. (2020). Evaluation of Quality of Life of Adult Patients with Celiac Disease in Argentina: From Questionnaire Validation to Assessment. Int. J. Environ. Res. Public Health.

[35] Harnett J.E., Myers S.P. (2020). Quality of life in people with ongoing symptoms of coeliac disease despite adherence to a strict gluten-free diet. Sci. Rep..

[36] Pratesi C.B.P., Häuser W., Uenishi R.H., Selleski N., Nakano E.Y., Gandolfi L., Pratesi R., Zandonadi R.P. (2018). Quality of life of celiac patients in Brazil: Questionnaire translation, cultural adaptation and validation. Nutrients.

[37] Falcomer A.L., Farage P., Pratesi C.B., Pratesi R., Gandolfi L., Nakano E.Y., Raposo A., Zandonadi R.P. (2021). Health-related quality of life and experiences of brazilian celiac individuals over the course of the sars-cov-2 pandemic. Nutrients.

[38] Pouchot J., Despujol C., Malamut G., Ecosse E., Coste J., Cellier C. (2014). Validation of a French version of the quality of life “celiac disease questionnaire”. PLoS ONE..

[39] Barzegar F., Pourhoseingholi M.A., Rostami-nejad M., Gholizadeh S., Malekpour M.R., Sadeghi A., Rostami K., Maleki I., Shahbazi S., Emami M.H. (2018). Transcultural Adaptation and Validation of Persian Version of Celiac Disease Questionnaire (CDQ); A Specific Questionnaire to Measure Quality of Life of Iranian Patients. Galen Med. J..

[40] Guennouni M., Admou B., Elkhoudri N., Bouchrit S., Ait A., Bourrahouat A., Krati K., Hilali A. (2022). Quality of life of Moroccan patients with celiac disease: Arabic translation, cross-cultural adaptation, and validation of the celiac disease questionnaire. Arab. J. Gastroenterol..

[41] Chaves C., Raposo A., Zandonadi R.P., Nakano E.Y., Ramos F., Teixeira-Lemos E. (2023). Quality of Life Perception among Portuguese Celiac Patients: A Cross-Sectional Study Using the Celiac Disease Questionnaire (CDQ). Nutrients.

[42] Moreno M.d.L., Sánchez-Muñoz D., Sousa C. (2022). Quality of Life in Teenagers and Adults with Coeliac Disease: From Newly Spanish Coeliac Disease Questionnaire Validation to Assessment in a Population-Based Study. Front. Nutr..

[43] Aksan A.A., Mercanligil S.M., Häuser W., Karaismailoğflu E. (2015). Validation of the Turkish version of the Celiac Disease Questionnaire (CDQ). Health Qual. Life Outcomes.

[44] Häuser W., Stallmach A., Caspary W.F., Stein J. (2007). Predictors of reduced health-related quality of life in adults with coeliac disease. Aliment. Pharmacol. Ther..

[45] Missbach B., Schwingshackl L., Billmann A., Mystek A., Hickelsberger M., Bauer G., König J. (2015). Gluten-free food database: The nutritional quality and cost of packaged gluten-free foods. PeerJ.

[46] Fry L., Madden A.M., Fallaize R. (2018). An investigation into the nutritional composition and cost of gluten-free versus regular food products in the UK. J. Hum. Nutr. Diet..

[47] Dietetic Association British (2021). Policy Statement: Gluten Free Food on Prescription.

[48] Peters M., Crocker H., Jenkinson C., Violato M. (2020). Withdrawing gluten-free food from prescriptions in England: A mixed-methods study to examine the impact of policy changes on quality of life. J. Hum. Nutr. Diet..

[49] Mogul D., Nakamura Y., Seo J., Blauvelt B., Bridges J.F.P. (2017). The unknown burden and cost of celiac disease in the U.S. Expert Rev. Pharmacoecon. Outcomes Res..

[50] Whitaker J.K.H., West J., Holmes G.K.T., Logan R.F.A. (2009). Patient perceptions of the burden of coeliac disease and its treatment in the UK. Aliment. Pharmacol. Ther..

[51] Truong V.A., Lang B., Conroy D.M. (2022). When food governance matters to consumer food choice: Consumer perception of and preference for food quality certifications. Appetite.

[52] Kreutz J.M., Heynen L., Arayess L., Vreugdenhil A.C.E. (2023). Celiac Disease and the Gluten Free Diet during the COVID-19 Pandemic: Experiences of Children and Parents. Medicina.

[53] King J.A., Jeong J., Underwood F.E., Quan J., Panaccione N., Windsor J.W., Coward S., deBruyn J., Ronksley P., Shaheen A.-A. (2020). Incidence of Celiac Disease is Increasing Over Time: A Systematic Review and Meta-Analysis. Gastroenterology.

[54] World Health Organization (WHO) (2019). Alphabetical List of WHO Member States.

[55] Ugur A. (2015). Public policies that can be implemented to struggle with celiac disease in Turkey. J. Econ. Soc. Thought.

[56] Smeets S.M., Kiefte-De Jong J.C., Van Der Velde L.A. (2022). Food insecurity and other barriers to adherence to a gluten-free diet in individuals with celiac disease and non-celiac gluten sensitivity in the Netherlands: A mixed-methods study. J. Clin. Gastroenterol..

[57] Sahin Y., Sevinc E., Bayrak N.A., Varol F.I., Akbulut U.E., Bükülmez A. (2022). Knowledge regarding celiac disease among healthcare professionals, patients and their caregivers in Turkey. World J. Gastrointest. Pathophysiol..

[58] Barzegar F., Rostami-Nejad M., Rostami K., Ahmadi S., Shalmani H.M., Sadeghi A., Khani M.A., Aldulaimi D., Zali M.R. (2019). Lack of health care professional’s awareness for management of celiac disease may contribute to under diagnosis of CD. Gastroenterol. Hepatol. Bed Bench.

[59] Violato M., Gray A., Papanicolas I., Ouellet M. (2012). Resource use and costs associated with coeliac disease before and after diagnosis in 3,646 cases: Results of a UK primary care database analysis. PLoS ONE.

[60] Norström F., Lindholm L., Sandström O., Nordyke K., Ivarsson A. (2011). Delay to celiac disease diagnosis and its implications for health-related quality of life. BMC Gastroenterol..

[61] Guennouni M., El Khoudri N., Bourrouhouate A., Hilali A. (2020). Availability and cost of gluten-free products in Moroccan supermarkets and e-commerce platforms. Br. Food J..

[62] MacCulloch K., Rashid M. (2014). Factors Affecting Adherence to a Gluten—Free Diet in Adults with Coeliac Disease. Paediatr. Child Health.

[63] Panagiotou S., Kontogianni M.D. (2017). The economic burden of gluten-free products and gluten-free diet: A cost estimation analysis in Greece. J. Hum. Nutr. Dietetics.

[64] Falcomer A.L., Santos Araújo L., Farage P., Santos Monteiro J., Yoshio Nakano E., Puppin Zandonadi R. (2020). Gluten contamination in food services and industry: A systematic review. Crit. Rev. Food Sci. Nutr..

[65] Farage P., Zandonadi R.P., Ginani V.C., Gandolfi L., Nakano E.Y., Pratesi R. (2018). Gluten-Free Diet: From Development to Assessment of a Check-List Designed for the Prevention of Gluten Cross-Contamination in Food Services. Nutrients.

[66] Miller K., McGough N., Urwin H. (2016). Catering Gluten-Free When Simultaneously Using Wheat Flour. J. Food Prot..

[67] De Oliveira P.M., Zandonadi R.P., Moreira A., Cutrim V., Nakano E.Y., de Queiroz F.L.N., Botelho R.B.A., Saraiva A., Raposo A. (2022). Eating Competence and Aspects Related to a Gluten-Free Diet in Brazilian Adults with Gluten-Related Disorders. Nutrients.

[68] Hall N.J., Rubin G., Charnock A. (2009). Systematic review: Adherence to a gluten-free diet in adult patients with coeliac disease. Aliment. Pharmacol. Ther..

