# Supplementary material for: From Struggle to Strength: A Multicentric Study on How Public Policies for Celiac Disease Transform Lives
Source: Nutrients. 2024 Aug 26;16(17):2855. doi: 10.3390/nu16172855 (PMC11396799; doi:10.3390/nu16172855)
Supplement: Supplementary file 1 [file nutrients-16-02855-s001.zip › nutrients-3170993-supplementary.pdf]

Table S1. Public Policies for Celiac Disease Score distribution per country.

Country	Year	Regulations concerning industrial food products	Regulations relating to meals	Specialized health service support;	Food allowance and/or financial incentive	Gluten-free certification for manufactured meals	Celiac disease associations	Total score
Argentina	2020	1	1	1	1	0	1	5
Argentina	2024	1	1	1	1	1	1	6
Australia	2020	1	1	1	1	1	1	6
Australia	2024	1	1	1	1	1	1	6
Brazil	2018	1	1	1	0	0	1	4
Brazil	2021	1	1	1	0	0	1	4
Brazil	2024	1	1	1	0	0	1	4
France	2014	1	1	1	1	1	1	6
France	2022	1	1	1	1	1	1	6
France	2024	1	1	1	1	1	1	6
Germany	2007	1	0	1	0	0	1	3
Germany	2024	1	1	1	1	1	1	6
Iran	2018	0	0	0	0	0	1	1
Iran	2024	0	0	1	0	0	1	1
Italy	2011	1	0	1	1	1	1	5
Italy	2013	1	1	1	1	1	1	6
Italy	2024	1	1	1	1	1	1	6
Morocco	2022	0	0	1	0	0	1	2
Morocco	2024	0	0	1	0	0	1	2
Portugal	2023	1	1	1	1	1	1	6
Portugal	2024	1	1	1	1	1	1	6
Spain	2022	1	1	1	1	1	1	6

Spain	2024	1	1	1	1	1	1	6
Turkey	2015	1	0	1	0	1	1	4
Turkey	2024	1	0	1	1	1	1	5
United Kingdom	2021	1	1	1	1	1	1	6
United Kingdom	2024	1	1	1	1	1	1	6
